# Supplementary material for: Are carbon emissions trading and green financial instruments synergistic? -Comprehensive quantitative research based on content analysis
Source: PLoS One. 2024 Mar 7;19(3):e0298601. doi: 10.1371/journal.pone.0298601 (PMC10919601; doi:10.1371/journal.pone.0298601)
Supplement: S1 Data — (ZIP) [file pone.0298601.s001.zip › Data/地级市Z_F工作报告.zip!/╔╜╢1⁄2/╝├─╧╩╨/└┤╫╘╣1⁄2╓┌║┼í╛┬φ┐╦╩2╛▌═°í┐_2004.docx]

各位代表：

　　现在，我代表市人民政府向大会报告工作，请予审议，并请各位政协委员和其他列席人员提出意见。

　　一、2003年全市经济和社会发展取得新成就

　　过去的一年，在省委、省政府和市委的正确领导下，全市人民高举邓小平理论伟大旗帜，认真实践"三个代表"重要思想，全面贯彻党的十六大精神，紧紧围绕"实现新跨越、建设新泉城"的奋斗目标，解放思想，干事创业，创新思路，团结奋进，经受住了国内外形势变化的新挑战和非典疫情的严峻考验，胜利完成了市十三届人大一次会议确定的各项任务，经济与社会发展取得新成就。

　　坚持"两手抓"，夺取了抗击非典与经济发展双胜利。去年上半年，面对突如其来的非典疫情，全市上下按照中央统一部署，沉着应对，靠前指挥，出台一系列有效措施和办法，形成指挥有力、运转高效的领导机制，构筑起全民预防非典的网络体系，实现了"堵住源头不输入，一旦输入不扩散，医护人员不感染，及时救治不死亡"的目标，赢得了抗击非典阶段性重大胜利。同时，毫不放松经济工作，千方百计变困难为机遇，变压力为动力，努力推动经济持续快速发展。实现国内生产总值1367.8亿元，增长14.5%，其中一产105亿元，二产599.6亿元，三产663.2亿元，分别增长5%、18%和13%。地方财政一般预算收入76.1亿元，按可比口径增长19.6%。超额完成了年初确定的工作目标。全市人民在"抗非"斗争中表现出的众志成城、万众一心的强大凝聚力和战斗力是我们战胜困难的强大动力。

　　结构调整力度加大，经济效益大幅提高。牢牢把握发展第一要务，加大改革创新力度，经济结构进一步优化，三次产业比重由8.2：41.8：50调整为7.7：43.8：48.5。农业结构稳步调整。粮食总产增长16.2%，附加值较高的蔬菜、畜牧产值占农业总产值的比重达到36.4%和33.8%；产业化经营水平明显提高，农民增收空间进一步拓宽，工资性收入占农民人均纯收入的比重提高到39.7%。二产效益明显提高。规模以上和销售收入过亿元的工业企业分别增长17.2%和29%；规模以上工业利税增幅比增加值增幅高20个百分点，亏损企业亏损额下降15.9%；经济效益综合指数达到148.8%，上升13.8个百分点，是"九五"以来最好水平；建筑业完成总产值239.1亿元，实现利税9.7亿元，分别增长22.7%和5%。三产结构趋好。实现社会消费品零售总额533.2亿元，增长15.6%，金融保险业、房地产业和其他服务业占三产比重分别提高了0.2、0.4和0.8个百分点；金融中心的作用增强，各项存款余额2449.5亿元，贷款余额2475.1亿元，分别比年初增长21.4%和18.3%。投资结构趋向合理。全社会固定资产投资504.9亿元，增长24.8%，投资率达到36.9%，提高3.2个百分点，有力地增强了经济发展的后劲。

　　大力培植"三个亮点"，发展活力进一步增强。大力推进高新技术产业化，加快科技创新体系建设。全市高新技术企业达到248家、产品333项，高新技术产业产值占规模以上工业总产值的比重为26%，提高2个百分点。创新领导机制，强化服务体系建设，招商引资取得新进展。实际利用外资4.47亿美元，增长20.6%；实际利用内资170亿元，增长41.7%；进出口总额20.2亿美元，增长35%，其中，出口8.4亿美元，增长20.2%。产权制度改革实现新突破，15户国有企业进行了规范化改制，1.2万名职工置换了国有身份，初步建立起国有资产管理体系框架。集中力量改善上市公司状况，渤海、小鸭成功复牌上市，轻骑、百货重组取得重大进展。非公有制经济发展速度加快，实现增加值482.7亿元，增长22.8%，占GDP比重达到35.3%，成为推动全市经济发展的重要力量。

　　城市规划建设实现重大突破，管理经营迈出新步伐。以贯彻省委常委扩大会议精神为契机，把握机遇，精心筹划，初步构建起符合市场化要求的城市规划、建设、管理、经营运行体系。在新一轮城市总体规划研究指导下，下大力量编制了东部产业带、东部新城、泉城特色风貌带、西部新城、西部片区等控制性详规及150多个重点区片城市设计和单项规划方案。全面展开"十大工程"建设，燕山立交、高架桥北延、纬六路道桥、环城公园治理二期、经六路西延等一批重点工程相继竣工；济南机场二期、经十路改造工程进展顺利；东部产业带14条道路工程全部完工，西部片区一期工程完成工程总量的60%。全市新增住宅建筑面积376.9万平方米，同比增长8.6%。城市气化率、供热普及率分别达到了94.6%和42%，提高1和2个百分点。全市新增绿地面积161万平方米，城市绿地覆盖率达到37.5%。大力开展城市环境综合整治，加大节水保泉力度，泉群恢复喷涌并达到近7年最高水位；城市公用服务体系投入运行，"12319"等服务热线为市民提供了便捷的综合服务。把经营城市的理念贯穿于规划、设计、拆迁、供地、建设、管理全过程，环卫、园林、公用事业等市场化改革迈出新步伐，加强土地"五统一"管理，土地市场秩序整顿取得明显成效。

　　政府职能转变加快，发展环境有所改善。以转变政府职能、创新管理体制和运行机制为突破口，从群众关心的热点问题入手，推出一系列改善发展环境的重大举措。制定了政府工作规则和深化行政管理体制改革10个方案，取消165个领导小组，规范了决策和工作程序。成立城市规划委员会、专家委员会和城市建设项目审批小组，完善城市重点工程建设指挥体系和城市规划控制管理体系，保证了城市规划建设有序推进。组建市政府资金结算中心，加强了政府性资金的统一调控和管理。赋予高新技术开发区43项市级管理权，变多口管理为一口委托管理。推进城市管理重心下移，完善"两级政府、三级管理、四级网络"的城市管理体系。成立行政执法局，相对集中和统一行使环卫、园林、规划、环保、工商、公安、城管、公用、人防、房管、建管、拆迁等部门的行政处罚权，"多头执法"问题得到解决。取消行政审批678项，占总审批项目的61%；取消行政事业性收费98项，占全部行政收费项目的20%。市成立发展环境投诉中心，建立外商投资全程代办制度。市及各县(市)建立了行政审批中心，实行联合审批、"一站式"服务，办事效率明显提高。推行重大决策事项公示、听证制度和各界代表对政府部门民主评议制度，推进了政府决策的科学化、民主化、规范化。

　　生活质量稳步提高，社会保障能力增强。城乡居民收入稳定增长。城市市区居民(非农业)人均可支配收入达到11013元，增长9.1%；农民人均纯收入3619.3元，增长7.9%；城乡居民存款余额758.3亿元，比年初增长17.8%。群众消费水平进一步提高。城市市区居民(非农业)人均消费性支出8395元，同比增长7.4%；居民消费价格指数为99.9%。城市居民人均住宅使用面积18.85平方米，农村居民人均生活用房面积32.5平方米，分别增加1.05平方米和1.9平方米。投资5.1亿元，完成"村村通"公路里程1676公里，使230个行政村新通了公路，72.2万农民直接受益；投资2800万元，实施农村供水工程，解决了263个村18.9万人的吃水困难问题。通过各种渠道安排就业再就业9.3万人，其中下岗失业人员再就业4.7万人，安置大龄就业困难人员1.2万人，劳务输出13万人，城镇登记失业率为3.82%。清欠农民工工资2.4亿元，接受城市居民最低生活保障6.72万人，城镇职工基本养老保险、失业保险制度进一步完善。

　　社会事业协调发展，精神文明建设取得新成果。深化科技体制改革，科技创新能力不断提高，全年取得重要科技成果226项，其中30%达到国际先进水平。教育事业有新发展，实施"五个一"优化升级工程，优质教育资源进一步扩大，教学质量明显提高，高考录取率高于全省平均水平，民办教育稳步健康发展，成人教育服务经济社会的功能增强。群众体育深入普及，继续保持全国先进水平；竞技体育获省级以上比赛金牌173.5枚，实现了历史性突破。文化体制改革迈出新步伐，艺术创作演出取得好成绩，共获省级以上奖励60余项，群众文化生活更加活跃，文化市场健康发展，文物保护与考古发掘取得新进展。计生工作纳入全市经济和社会发展总体规划，圆满完成年度人口责任目标，人口自然增长率控制在5‰以内。新闻出版、广播电视、社会科学、档案管理、外事侨务、民族宗教、妇女儿童、老龄、残疾人等事业都有了新的发展。政府依法行政水平进一步提高，全年办理人大代表批评和意见329件、政协提案480件。廉政建设和反腐败斗争逐步深入，审计监督作用得到较好发挥，纠正部门和行业不正之风不断取得新成效。牢固树立"济南稳则全省稳"的省会意识，深入开展严打斗争和安全生产专项整治活动，加强基层基础工作，积极预防、疏导和化解社会矛盾，人民群众安全感进一步增强，社会治安秩序和安全生产形势持续稳定。广泛开展"双拥"共建活动，国防教育、征兵工作和国防后备力量建设不断加强。群众性精神文明创建活动广泛开展，形成了稳定健康、奋发向上的社会环境。

　　各位代表，过去一年各项工作成绩的取得，是省委、省政府和市委正确领导的结果，是人大、政协的监督支持和全市人民努力奋斗的结果，是驻济部队、各界人士积极参与的结果。在此，我代表市政府向辛勤工作在各行各业的全体市民，向给予政府工作大力支持的各级人大代表、政协委员、各民主党派、工商联和各界人士，向关心帮助地方工作的驻济人民解放军和武警官兵，向为济南发展做出贡献的港澳台同胞、海外侨胞和国内外朋友们表示崇高的敬意和衷心的感谢！

　　各位代表，过去一年取得的成绩令人鼓舞，但前进道路上还有不少矛盾和问题。主要表现是：经济结构战略性调整步伐不快，结构性矛盾、区域性矛盾仍然比较突出；市场取向的改革滞后，体制性、机制性障碍较多，市场配置资源的基础性作用还未得到充分发挥；非公有制经济比重仍然偏低，外向型经济还很薄弱；发展环境仍然不够宽松，社会信用和市场秩序有待于进一步规范；就业压力较大，农民收入增长缓慢，城乡弱势群体生活困难和保障问题还需下大气力解决；政府转变职能还不到位，行政效率不高、执法行为不规范的现象依然存在。我们应正视这些矛盾和问题，立足长远，着眼当前，千方百计调动各方面力量，坚持用改革和发展的办法努力加以解决。

　　二、今年政府工作的主要目标和任务

　　2004年是实现"十五"计划的关键一年，也是深化改革、扩大开放、促进发展的重要一年。在新的一年里，政府工作的指导思想是：以邓小平理论和"三个代表"重要思想为指导，全面贯彻党的十六大和十六届三中全会精神，紧紧围绕"实现新跨越、建设新泉城"的奋斗目标，牢固树立全面、协调、可持续的发展观，坚持以人为本、统筹兼顾，保护好、引导好、发挥好各方面解放思想、干事创业的积极性，继续实施科技、产业、制度、环境"四个创新"，以结构调整为主线，以改革开放为动力，以转变政府职能、优化发展环境为重点，实现工业强市、县域经济、城市现代化、三个亮点(高新技术、民营经济、外向型经济)和现代服务业的新突破，促进经济持续快速协调健康发展和社会全面进步。

　　今年全市经济社会发展的主要预期目标是：国内生产总值增长14%；地方财政一般预算收入按可比口径增长15%；社会消费品零售总额增长14%；非公有制经济增加值增长30%；外贸出口增长14%，实际利用外资增长40%；全社会固定资产投资增长30%；城市市区居民(非农业)人均可支配收入增长8%，农民人均纯收入增长5%；城镇登记失业率控制在4.5%以内；人口自然增长率控制在6‰以内。

　　实现以上目标，在工作指导上努力把握好以下几点：一是坚持加快发展不动摇。全市上下要始终把加快发展作为富民强市的第一要务，营造聚精会神搞建设、一心一意谋发展的浓厚氛围，以思想更解放一些、改革力度更大一些、付出努力更多一些的胆识和魄力，在调整中抢抓机遇，在竞争中打破平衡，在跨越式、超常规发展中缩小与先进地区的差距。二是坚持全面、协调、可持续的发展观。把科学的发展观作为经济工作必须长期坚持的重要指导思想，作为解决经济社会发展中诸多矛盾必须遵循的基本原则落实到各项工作中，促进城乡经济、区域经济、经济与社会事业、人与自然环境协调发展。三是坚持用改革、创新和市场的办法解决问题。遵循经济发展规律和市场法则，积极探索和研究从根本上、整体上解决问题的办法，进一步突破束缚生产力发展的体制性、机制性障碍，努力创造良好发展环境。四是坚持从实际出发，狠抓关键环节。着眼于提高济南经济的综合竞争力，把推进结构调整作为提高经济整体素质的关键，把工业强市作为支撑经济发展全局的战略重点，把培植"三个亮点"作为加快发展的突破口，把搞好城市规划建设管理作为提升城市综合竞争力的重大举措，把加大投资力度作为增强发展后劲的根本保障，强化综合措施，形成集聚效应，创新发展优势。五是坚持把实现好、维护好人民群众的根本利益作为处理改革、发展、稳定关系的出发点和落脚点，真正做到权为民所用、情为民所系、利为民所谋，把改革的力度、发展的速度和社会可承受的程度有机结合起来。六是坚持狠抓落实、务求实效的作风。弘扬艰苦奋斗、真抓实干的精神，不搞形式主义，不做表面文章，堂堂正正做人，踏踏实实办事，埋头苦干，攻坚破难，逐项逐件抓好落实。

　　三、突出以优化结构为主线，努力提高经济整体竞争力

　　围绕提高综合竞争力，大力培植支柱产业，改造提升传统产业，积极发展新兴产业，努力构筑具有济南比较优势、富有竞争力的产业体系。

　　以农民增收为中心，促进农业和农村经济发展。全面建设小康社会，必须坚持城乡一体，全面推进县域经济发展。要高度重视"三农"问题，围绕农民增收，加快推进农业产业化、农村工业化和城镇化，全面落实统筹城乡发展的各项政策。继续调整优化农业结构。高度重视粮食安全，正确处理结构调整与保护耕地和粮食生产的关系，大力发展优质粮油品种，提高单产，稳定总产。以组织实施"双十工程"为抓手，大力发展特色、优势、高效、创汇产业，加大政策和资金扶持力度，推动优势和特色产品向产区集中，提高蔬菜、牧业、林业占农业总产值的比重。加快农业产业化步伐。鼓励工商企业投资发展农产品加工业和营销，培育壮大一批有带动能力的龙头企业，组织引导农民在民主、自愿的基础上发展专业经济合作组织，提供产前、产中和产后服务。加强农产品标准化建设。建立健全农产品质量标准体系、检验检测体系和认证体系，大力实施农产品无公害生产，建成一批管理严格的绿色食品和有机食品生产基地，提高以市农高区为龙头的农业科技示范园区建设水平。加强农业基础设施建设和生态环境建设。进一步加大农业投入，抓好引黄工程与"济平干渠"联接、卧虎山水库续建工程等设施建设，加强山区综合开发和扶贫开发，继续搞好"村村通"公路建设，推进通讯、供电、供水、信息网络更快地向农村延伸覆盖，提高农村基础设施配套水平。进一步落实农村经济政策。稳定土地承包制度，严格遵循"依法、自愿、有偿"的原则，引导规范土地承包经营权合理流转。深化农村税费改革，降低农业税税率1个百分点，落实对粮农间接补贴为直接补贴的办法，加强农村"两工"管理，规范"一事一议"，切实减轻农民负担。完善农村富余劳动力向二、三产业和城镇转移的有关政策，加快农村城镇化进程，加强对农村劳务输出的组织引导，拓展农民增收空间。

　　以推进"2133"工程为重点，全力实施工业强市战略。动员全社会力量"大抓工业、抓大工业"，以结构调整为主线，以产业优化升级为目标，以加强载体建设为依托，大力推进新型工业化进程。全力抓好"2133"工程。按照规划要求，明确各级责任，层层分解目标，加大投资力度，落实扶持政策，重点抓好一批合资合作、技术改造、规模扩张和搬迁改造项目，确保全年规模以上工业完成投入260亿元、实现销售收入1500亿元、发展规模以上企业300家、销售收入过亿元的企业30家。培植高新技术产品。采取研发、培育、引进等各种措施，突出发展科技含量高、核心竞争能力强、市场份额大的优势产品，并努力把优势产品培育成国内外的知名品牌。壮大骨干企业群体。坚持扶优扶强的方针，依托优势名牌产品，运用经济手段、政策手段、市场手段，引导生产要素向优势企业集聚，迅速形成一批销售收入过10亿元的骨干企业，促进重汽、济钢、浪潮、炼油、将军、力诺、化纤、山水、锅炉、松下等一大批企业成为带动工业经济发展的龙头企业。打造优势产业链条。在深入调研论证的基础上，搞好产业链的规划和实施。要以龙头企业为支撑，以优势产品为纽带，以合理布局为基础，以科学管理为保障，形成上下游产品有机连接、配套互动的产业格局，尽快形成一批具有竞争优势、体现济南特色的产业链条。强化工业载体建设。以规划为龙头，以设施建设为基础，以配套服务为核心，以项目落地为根本，加快载体建设步伐。各类开发区要与城市发展规划和土地利用规划有机统一，按照高标准建设、高效能管理的要求，搞好基础设施配套，吸引更多"三高"项目进区建设，使各类开发区真正成为产业的集聚区、生态建设的示范区、科技人才的集聚区、体制创新的先行区。

　　以现代服务业为重点，推动第三产业实现新突破。以扩大内需、扩大就业、扩大财源为目标，统筹规划，重点突破。加快改造提升商贸流通等传统服务业。抓紧制定商贸业布局规划，进一步增强和发挥城区中心商业区和五个区域性商贸中心的带动作用，鼓励国内外知名商业企业和强势流通企业通过连锁经营、电子商务等新型业态实现规模扩张，带动传统商贸流通业提升改造，扩大对省内外辐射能力。大力发展旅游业。按照做足需要、做强产品、做大卖点的思路，充分运用泉水喷涌良机，加大策划力度，着力打造以趵突泉、大明湖为重点的泉城特色标志旅游品牌，规划整合一批专项旅游路线，引进新建一批具有国际影响力的大项目，加大旅游市场推介和促销力度，积极组织好黄金周、亚洲杯足球赛、摩托艇世锦赛等一批节庆、赛事期间的旅游活动。高起点地发展现代物流业。充分发挥经济、交通、信息网络优势，统筹规划布局，引进国内外知名物流企业，培育壮大骨干物流企业，积极发展第三方物流，加快物流资源整合，推进物流园区建设，构筑区域性物流中心。积极发展金融保险业。制定实施区域性金融中心发展规划，健全金融市场体系、创新金融工具，推进对外开放，争取外资金融、保险机构和外地金融机构总部进入济南市场，进一步增强我市集金融管理、保险、证券、典当、拍卖、信托、融资、结算、服务和信息枢纽于一体的跨省际金融中心的综合服务功能。加快发展会展业等新兴产业。按照市场规律运作好各类会展活动，努力打造一批享誉国内外的会展品牌。积极发展房地产、信息服务、中介服务等新兴都市型服务产业，大力发展各类便民、利民的社区服务业。结合错时工作制，在交通、餐饮、商业、文化、体育等方面推出方便和丰富市民夜生活消费的一些新举措，推动第三产业的发展。

　　四、大力培育"三个亮点"，实现高新技术、民营经济和外向型经济新突破

　　发展高新技术、民营经济和外向型经济，是增强市场竞争力和发展活力的重大战略举措，要审时度势，克服困难，创造性地开展工作，使"三个亮点"真正亮起来。

　　以增强产品核心竞争力为重点实现高新技术产业新突破。重点培育壮大电子信息、生物制药、新材料三大产业，全年新增高新技术企业100家，高新技术产品100项，高新技术产业产值占规模以上工业总产值的比重提高2个百分点。积极运用先进适用技术改造提升传统产业，加快信息化带动工业化步伐，大力实施制造业信息化工程，加快火炬计划产业基地建设，培植壮大软件及系统集成骨干企业，集中扶持机械、建材、化纤纺织等3个示范行业和20家示范企业，加速我市制造业的跨越式发展。集中力量加快以高新技术开发区为重点的各类科技园区建设，重点推进齐鲁软件园、力诺工业园、出口加工区的建设，结合东部产业带的开发建设，打造高新技术产业发展基地。加强对高新技术企业发展的引导，健全专家咨询网络，建立完善高新技术投融资机制，不断加大政府科技投入，鼓励企业、民间和境外资本开展风险投资。

　　以增强经济发展活力为重点实现非公有制经济新突破。按照"政治平等、政策公平、法律保障、放手发展"的方针，坚持政策启动、改革推动、骨干带动、科技拉动，实现非公有制经济增加值增长30%以上。进一步放宽非公有制经济市场准入领域。扫除一切制约发展的思想障碍，废除一切限制发展的歧视性政策，革除一切落后的管理手段和行为，在市场准入、监督管理上一视同仁，营造公平的政策环境和市场环境，让非公有制企业在投融资、税收、土地使用和对外贸易等方面与其他企业享受同等待遇，鼓励民间资本以多种形式进入城市公用事业和基础设施建设、运营和经营。鼓励有条件的骨干民营企业做大做强。扶持主业突出、管理先进、核心竞争力强的优势非公有制企业，参与国有、集体企业的改制改组，在资金、电力、土地、项目等方面创造条件、重点倾斜。创造良好的外部环境。建立健全信用担保、诚信评估、人才培训、法律服务等体系，努力解决民营企业融资难的问题，努力使民营经济成为我市经济最具活力的最大增长点。

　　以提高利用外资规模和水平为重点实现外向型经济新突破。统筹国内开放和对外开放，充分利用两个市场、两种资源，积极扩大利用外资规模，确保实际利用外资增长40%以上，实际利用内资增长30%以上。拓宽对外开放领域。紧紧围绕全市产业结构调整，以加工制造业、城市基础设施和服务业为重点，拓展金融、保险、旅游、教育、文化、体育、卫生等各领域，全方位扩大开放，宽领域招商引资。做好项目策划推介工作。加强重大项目的策划、论证和包装，筛选一批大项目、好项目进市及各县(市)区的项目库。通过多种形式有计划、有重点地搞好项目推介，集中组织好赴韩、日、港、台的大型招商活动。提高利用外资的质量和水平，抓好市内大企业与跨国公司的战略合作，重点抓好一批千万美元以上大项目的引进工作。加大项目落地协调力度，建立重点项目协调联席会制度，协调解决遇到的难题。努力扩大出口贸易。优化出口产品结构，大力提高机电产品、高新技术产品出口比重，加快发展钢铁制品、机电产品、纺织服装、农副产品出口。实施"走出去"战略。鼓励有条件的企业到境外投资办厂，对外开展工程承包、劳务输出。积极克服国家出口退税政策调整带来的影响，主动应对国外贸易壁垒，推动外贸出口迈上新台阶。

　　五、大力推进城市规划建设管理经营，努力创造优良人居环境

　　抓住新一轮城市发展的战略机遇期，坚持"四高"方针，按照"新区开发，老城提升，两翼展开，整体推进"的思路，集中力量抓好完善规划体系、重点工程建设，创新城市管理体制和经营机制。

高起点深化以规划体系为重点的城市规划。完善以总体规划、分区规划、近期建设规划、控制性详规、修建性详规为主要内容的城市规划体系，搞好城市生产力和社会事业发展规划，加快编制产业、交通、教育、卫生、名城保护等专业规划，统筹城市空间布局。引进国内外一流规划设计力量，发挥城市规划委员会和专家组的作用，提高规划设计水平，提高项目审批效率和质量。强化城市规划管理和执法，任何单位和个人不得违反城市规划乱插乱建，违者必须受到严肃处理。各县(市)要围绕加快新一轮发展，充分做好与中心城市借势发展和对接的文章，高标准搞好产业布局以及县城及重点建制镇的规划建设，引导村庄建设，完善功能，提升形象，形成与中心城市功能互补、规模适度的中小城市(镇)。

　　高标准完成以道路建设为重点的城市基础设施建设。加快推进交通、管网、绿化、供水、设施、康居建设，名城、蓝天保护，河道、容貌整治"十大工程"。重点抓好东部产业带和西部片区的道路新建工程，开工建设济南至菏泽、济南至莱芜高速公路和绕城高速公路跨越黄河的大北环和黄河三桥；按照"三拆、二改、一建"的要求，完成经十路、经一路、舜耕路-青年东路-湖山路、济洛路、济王路、工业南路、104国道的综合整治工程；抓好一批市区主要道路的铺装、维修改造工程。按照截污、清淤、增绿、造景、蓄水的要求，实施玉绣河、杨家河、大沙河的河道整治工程，做好小清河等河道整治的前期准备。完成济南机场航站区二期工程、体育中心建设工程、大明湖综合整治一期工程、燕山健身广场建设工程，实施植物园、泉城广场周边综合整治工程。各项工程要在城市重点工程建设指挥部的统一领导下，按照市场经济规律和现代工程管理要求，认真落实项目法人制、专家评审制、招标投标制、工程监理制、政府采购制、财务核算制和工程审计制，严格控制工期、工程质量、工程造价，确保实现精品工程、效益工程、廉政工程、民心工程的目标，经得起社会评价和历史检验。

　　高效能搞好以生态建设为重点的"创模"和环境综合整治。以创建国家环保模范城为目标，加大环境保护和生态建设力度。推动生态工业产业链建设，控制工业固体废物排放总量，建成济南市医疗废物处置中心。积极推广清洁能源，加强对燃煤污染、机动车尾气、扬尘、粉尘、油烟等污染防治，加快污水处理厂及管网建设。加大节水保泉和黄河、小清河保护力度，大力实施荒山绿化、退耕还林、平原绿化、绿色通道建设等绿化建设工程。集中力量清理违章建筑和违规建设，重点治理局部区域"脏乱差"问题，全面推行辖区管理目标责任制，以整洁有序的形象迎接建国55周年。

　　高水平实施以优化资源配置为重点的城市经营。积极运用市场机制，广泛吸引社会资金，形成政府引导、社会参与、市场运作的多元化投融资格局。充分利用城市基础设施等有形资产和各种无形资产，通过公开拍卖和转让经营权、冠名权、广告权和管理权，实现城市资源效益最大化。按照土地规划、征用、整理、出让、管理"五统一"的要求，搞好所有改造和新建道路两侧可利用地块的整体策划和开发招商，努力实现城市建设资金自求平衡、滚动发展、良性循环。

　　六、加大改革和投入力度，努力增强经济发展活力和后劲

　　围绕完善社会主义市场经济体制，消除经济社会发展中的体制障碍，有步骤有重点地推进各项改革。继续加大投资力度，优化投资结构，提高投资效益。

　　积极推进和深化各项改革。加快产权制度改革步伐。大力发展混合所有制，全面推进工业、农业、商业、城建、外贸等领域的股份制改造，完善公司法人治理结构，健全企业激励约束机制，建立归属清晰、权责明确、保护严格、流转顺畅的现代产权制度。按照分口负责、配套改革、自求平衡的原则，加快国有企业改革步伐，争取市属工业企业国有资本降到50%以下，商贸企业降到30%以下。积极稳妥地推进困难企业的重组解困工作。加快国有资产管理体制改革。按照权力、义务和责任相统一，管资产、管人、管事相结合的原则，加快组建市国有资产管理机构，完善授权经营制度和监管机制。加快事业单位的综合配套改革，剥离事业单位的政府管理职能，逐步建立起与市场经济体制相适应的管理体制、人事和分配制度。加快城市供水、供热、供气、公交等公用事业的市场化进程。加快投融资体制改革。健全政府投资决策和项目法人约束机制，切实确立企业的投资主体地位，实现谁投资、谁决策、谁受益、谁担风险。加快财税体制改革。按照"税收属地征管，收入格局基本稳定，调动市县(区)两个积极性，确保收入稳定增长"的思路，调整和完善市、县(市)区财税体制。以预算编制改革为核心，协调推进国库集中支付、政府采购和收支两条线改革。加大对农村和农业的财政支持，大力推进社会综合治税，彻底清理税收优惠政策，依法加强行政性收费和基金的征收。健全公共财政体制，统筹使用政府公共资金。严格控制财政支出，按照"保工资、保稳定、保法定支出"的顺序安排支出，大力减少一般性支出。整顿和规范市场经济秩序。大力推进市场开放，促进商品要素自由合理流动，完善行政执法、行业自律、舆论监督、群众参与相结合的市场监管体系，严厉打击制假售假、商业欺诈等违法行为，做好文化、烟草、集贸市场和旅游市场及危险化学品的专项整顿工作，适时组织好第一次全国经济普查。加快发展土地、技术、劳动力等要素市场，规范产权交易。加快建设企业和个人信用服务体系，尽快形成以道德为支撑、产权为基础、法律为保障的社会信用制度，构建"诚信济南"。

　　优化投资结构，增强对经济增长的拉动作用。把扩大固定资产投资作为培育经济增长点、调整经济结构、加快经济发展的重大举措。努力拓宽社会投资和企业融资渠道，保持投资较快增长，完成全社会固定资产投资650亿元，实现全社会固定资产投资占国内生产总值、工业投资占全部投资、引进资金占全部投资的40%以上的目标。强化目标责任，坚持"市外就是外"，一二三产业一起上，市、县(市)区联动，精心筹划和包装一批项目。把握好资金投向。重点加大高新技术、高附加值、高出口创汇、高税收产品的投入，统筹安排好基础设施的投入，适当加大公共产品的投入，控制低水平重复建设，禁止耗能高、污染大、资源浪费严重项目的投入。拓宽资金渠道。坚持扩大招商引资、增加金融信贷资金、争取国家和省专项资金、拓宽经营城市门路、加强资本市场融资、吸引民间投资同时并举。加强重点项目调度，特别是对投资亿元以上的大项目，有关部门要及时解决遇到的各种实际问题，促进早开工、早投产、早见效。

　　七、推动社会事业全面进步，加强精神文明和民主法制建设

　　树立以人为本和全面、协调、可持续的发展观，推动各项社会事业全面发展，努力把社会主义精神文明和民主法制建设提高到新的水平。

　　积极推进科教文化事业加快发展。以创建人力资源优势为目标，继续实施科教兴市和人才强市战略。进一步深化科技管理体制改革，加快建设以高等院校和重点科研机构为主体的知识创新体系、以企业为主体的技术创新体系、以各类科技中介服务组织为主体的科研成果推广应用体系，提高我市科技自主创新持续发展能力。教育工作要深化改革，优化结构，调整布局，提高质量，实现优质教育资源均衡发展，确保全市基本普及高中阶段教育；整合职业教育，发展成人教育，加强农村教育，支持民办教育，鼓励多种模式办学和国际合作办学。把人才资源开发纳入经济和社会发展整体规划。动员和集中全社会的力量和资源，多层次培养、多渠道引进、大规模开发大批适应现代化建设的各类人才。以社会效益为主要目标，加快文化体制改革。拓宽市场运作渠道，增加对公益性文化事业的财政投入，整合文化资源，逐步建设一批代表省会城市、历史文化名城形象的精品文化设施工程和历史文物保护工程。发展和培养名牌演艺团体，创作生产更多体现泉城特色、积极健康和人民群众喜闻乐见的文化产品和精品剧目，繁荣发展文化市场，促进文化事业和文化产业的协调发展。积极发展体育事业。深入开展全民健身活动，大力发展竞技体育，高水准、高效益办好亚洲杯足球赛，加大体育中心公园、体育主题广场等基础设施的筹划和建设力度。推进公共卫生事业改革与发展。深化卫生体制改革，加快卫生资源整合，建立健全农村、社区卫生服务体系和合作医疗制度。加强疾病预防控制体系和医疗救治体系建设，搞好市疾病预防控制中心和市传染病医院建设工程。建立健全公共卫生长效机制和突发公共卫生事件预警、应急机制。健全卫生监管体系，保证群众的食品、药品和医疗安全。开展计划生育优质服务，创新工作运行机制，稳定低生育水平，促进优生优育，确保实现计划生育各项目标。积极做好对台、外事、侨务、民族、宗教、妇女、儿童、老龄、残疾人等工作，推动新闻出版、广播电视、社会科学、档案管理等各项社会事业的发展。

　　加强精神文明和民主法制建设。认真落实《公民道德建设实施纲要》，完善全市创建全国文明城市领导体系和工作机制，深入开展创建文明社区、文明行业、文明村镇和争做文明市民活动。继续开展科教、文体、法律、卫生、交通安全"五进社区"活动，努力打造学习型全民教育网络。深入开展以"诚实守信，文明服务"为主题的"行风万人评"活动，进一步推行社会服务承诺制。继续做好拥军优属工作，落实优抚安置政策，积极支持国防建设。加强法制宣传教育，深化"四五"普法活动，全面提升以法治市水平，推进地方、行业、社区和基层依法治理，逐步推开社区居委会换届直选活动。开展创建"平安济南"活动，按照"重心下移、强化基层、落实责任"的方针，运用市场手段建设基层平安机制，提高社会整体防范能力。完善和落实安全生产各项政策措施，加强监督管理体系建设，继续开展安全生产专项整治，认真排查各种事故隐患，防止重特大事故发生。高度重视人民群众来信来访工作，加强基层矛盾纠纷调处、预防和化解各类不安定因素，促进社会安定团结。

　　八、健全就业和社会保障体系，努力解决群众关心的热点问题

　　就业是民生之本，也是安国之策。各级政府要把扩大就业、提高群众生活水平放在更加突出的位置，着力解决群众生产生活中的突出问题。

　　突出抓好就业和再就业。要健全就业再就业工作责任制，把新增就业和城镇登记失业率目标纳入各级政府考核体系。要大力开拓就业领域和渠道，把深化改革、促进发展、调整结构和扩大就业有机结合起来，完善劳动者自主择业、市场调节就业、政府促进就业的机制。抓好加快发展劳动密集型产业、大力发展服务业和中小企业以及个体私营经济等政策的落实。拓展和开发商贸流通、餐饮服务、旅游信息、公共卫生、家政服务等新型服务业和公益性就业岗位。加大再就业投入，健全再就业援助制度，加强职业教育、技能培训和企业培训。大力实施就业困难群体帮扶工程，充分发挥街道(镇)和社区劳动保障服务工作机构的作用，突出解决好"4050"人员和一户双代、夫妻双方、抚养未成年子女和单亲家庭以及企业军转干部再就业困难户的再就业问题。

　　建立健全社会保障体系。继续巩固"两个确保"，搞好"三条保障线"的衔接，建立社会保险基金预算制度，依法加强社会保险费的征缴和清欠工作，扩大企业基本养老保险覆盖面，推进失业保险、城镇职工基本医疗保险、职工工伤和生育保险制度，落实城市居民最低生活保障制度。把符合条件的人员纳入低保，做到应保尽保。加强社区服务平台建设，基本实现企业退休人员社会化管理服务，确保离退休职工养老金和下岗职工基本生活费按时足额发放。建立与现代产权制度相适应的收入分配制度，积极稳妥地推行国有企业经营者年薪制，适时调整企业最低工资标准，加强对垄断行业收入分配的监管，规范分配秩序。

　　切实解决群众关心的难点问题。建立和完善失地农民的生活保障机制，继续清理对农民工的歧视性政策，加大对拖欠农民工工资问题的检查和处罚力度，确保进城务工子女依法接受义务教育。加大对农村扶贫、困难乡镇及乡村公路建设的扶持力度，完成"三农"投入2.2亿元，同比增长70%。完成农村学校危房改造任务，加快农村饮水解困工程建设，努力解决历史性缺水人口的饮水问题。解决好农村特困户的基本生活，完善农村五保户生活保障制度，确保供养资金及时足额发放。妥善解决城市特困家庭"上学难、看病难、就业难、住房难"等实际问题，切实解决好城市建设中旧城改造、拆迁安置问题。加强赈灾救济工作，大力发展社会互助事业，切实解决鳏、寡、孤、独、残等弱势群体的生产生活问题，积极开展法律援助。对侵害群众利益、群众反映强烈的问题，要严厉查处，决不姑息。

　　九、推进政府职能转变，提高执政能力和依法行政水平

　　各级政府要把职能尽快转变到为市场主体服务和创造良好发展环境上来，以崭新的面貌和扎实的作风开创经济和社会发展的新局面。

　　切实解决政府管理越位、缺位和错位问题。按照经济调节、市场监管、社会管理和公共服务的要求，把该由市场调节的还给市场，该由企业决定的还给企业，更大限度地发挥市场配置资源的主体作用。加快建立行为规范、运转协调、公正透明、廉洁高效的行政管理体制。下决心撤销、裁减、合并那些不适应政府职能转变、名存实亡、机构臃肿、职能交叉的部门和事业单位，合理配置行政权力，减少人员编制，提高行政效能。继续推进政企分开、政资分开和政事分开，培育和规范社会中介组织，运用经济和法律手段调节经济，进一步降低行政成本，健全完善经济运行统计和监测体系，加强经济调控导向的功能互补和信息共享。继续深化城市管理重心下移改革，落实区、街管理责任。充分履行好社会管理和公共服务职能，努力提供优良公共产品。

　　严格做好行政许可各项清理工作。以实施《行政许可法》为契机，修改和废止市政府规章和规范性文件设定的行政许可，依法清理纠正不符合规定的行政许可主体。清理行政许可收费，任何行政机关不得收取法律、行政法规允许外的任何费用。加快推行相对集中行政许可制度，建立健全行政许可的规范性文件、重大行政许可决定备案制度、申诉检举制度、统计制度。规范保留的行政许可事项，集中到市政府审批中心，实行"一门受理、联合审批、规范收费、限时办结"的"一条龙"服务，推进以电子政务为支撑的网上审批，从体制上、制度上、根本上解决办事效率低、服务质量差等问题。

　　努力提高政务公开和依法行政水平。完善科学决策机制，把科学决策、民主决策、依法决策有机统一起来，进一步完善重大事项集体决策制度、人民建议征集制度、专家咨询制度、社会公示和社会听证制度，建立政府发言人制度，定期向社会公布政务信息，增强群众对政府工作的知情权、参与权和监督权。认真执行人大及其常委会的决议、决定，自觉接受人大的法律监督和政协的民主监督，办理好人大代表建议和政协提案。全面推行行政执法责任制、评议考核制、行政效能告诫和责任追究制度，履行行政复议、行政赔偿职责。进一步扩大行政综合执法，切实解决好执法部门职责交叉、多头执法、重复处罚、执法扰民等问题。全面落实"优化发展环境监督检查年"的各项措施，建立优化发展环境民主评议长效机制。强化市发展环境投诉中心职能，建立健全投诉、监督机制，切实解决好企业的各种困难和问题，营造亲商、富商、安商的创业环境。

　　切实加强廉政建设和反腐败工作。坚持标本兼治、综合治理，加大从源头上预防和治理腐败，从体制、机制、法制"三位一体"上推进反腐倡廉工作，重点抓好领导干部廉洁自律、查办大案要案和纠正部门、行业不正之风三项重点任务。进一步推进行政审批、财政管理和干部人事三项制度改革，严格加强土地批租、工程招标、产权交易、政府采购等关键环节的监管。充分发挥审计监督作用，强化对重点领域、重点部门、重点资金的审计监督，搞好领导干部离任经济责任审计，实行审计结果定期通报制度。继续抓好政府机关处科级干部廉洁勤政行为规范的落实和考评，强化监督制约机制。

　　发扬狠抓落实的工作作风。圆满完成今年的各项任务，关键在于狠抓落实。各级领导干部，要以广大群众的根本利益作为一切工作的出发点和落脚点，牢固树立"两个务必"的思想，把心思和精力用在为民谋利益上。大力弘扬求真务实、讲求实效的作风，坚持一切从实际出发，着眼于解决发展中存在的实际问题。坚持实践第一的观点，与时俱进，大胆探索，敢于创新。大兴调查研究之风，深入基层，深入群众，特别是深入到困难多、问题多、矛盾多的地方，倾听群众呼声，关心群众疾苦，切实为基层、为群众排忧解难。进一步精减会议，压缩文件，切实改进会风、文风。建立严格的工作目标责任制，抓重点、抓关键、抓调度、抓考核，确保各项工作落到实处。

　　各位代表，加快推进我市经济和社会发展，是全市人民的共同愿望，是各级政府义不容辞的责任和使命。我们要更加紧密地团结在以胡锦涛同志为总书记的党中央周围，在省委、省政府和市委的领导下，与时俱进，迎难而上，抢抓机遇，干事创业，努力夺取全市改革开放和社会主义现代化建设的新胜利，以优异的成绩向建国55周年献礼!
